# The Role of Mg Content and Aging Treatment on the Tensile and Fatigue Properties of Die-Cast 380 Alloy

**DOI:** 10.3390/ma15248844

**Published:** 2022-12-11

**Authors:** Agnes M. Samuel, Yasser Zedan, Ehab A. Elsharkawi, Mohamed H. Abdelaziz, Fawzy H. Samuel

**Affiliations:** 1 Department des Sciences Appliquées, Université du Québec à Chicoutimi, Chicoutimi, QC G7H 2B1, Canada; 2 Department of Mechanical Engineering, École de Technologie Supérieure (ÉTS), Montreal, QC H3C 1K3, Canada; 3 Division of Engineering, Saint Mary’s University, Halifax, NS B3H 3C3, Canada; 4 Département PEC, Université Française d’Égypte, Ville Shorouk, Le Caire 4923116, Egypt

**Keywords:** die-cast 380 alloy, effect of Mg content/aging treatment, tensile and fatigue properties

## Abstract

The main objective of this contribution was to determine the impact of magnesium (Mg) concentration and solidification rate (about 800 °C/s) on the mechanical properties of commercial A380.1 die-cast alloy. Respective amounts of 0.10%, 0.30%, and 0.50% Mg were used to establish their influence on the main tensile properties, namely, the ultimate limit, the elastic limit, and the percentage of elongation to fracture. The study also focused on the effect of magnesium on the fatigue behavior of A380.1 alloy where the role of surface defects and internal defects (porosity, oxide films, and inclusions) on the alloy fatigue life was also determined. The tensile properties were analyzed in order to optimize the heat treatments of T6 (under-aging) and T7 (over-aging). Consequently, the influence of several parameters was evaluated using tensile testing and optical and scanning electron micrography. Fatigue strength was investigated by performing rotational bending tests. The results show that the alloy tensile strength parameters improve with up to 0.3% Mg. Further addition of Mg, i.e., 0.5%, does not produce any significant improvement with respect to either traction or fatigue. It is observed that the tensile properties fluctuate according to the Guinier–Preston zones which occur during heat treatment, while the fatigue properties decrease as the Mg content increases. In contrast to a mechanical fatigue failure mechanism, in the present study, cracks were initiated at the sample’s outer surface and then propagated toward the center.

## 1. Introduction

From mass-market vehicles such as the Ford F-150 to luxury cars such as Audi, Mercedes Benz, and Land Rover, aluminum is increasingly the “material of choice” for automakers thanks to its strength and environmental advantages. Current interest in the use of aluminum in automotive applications includes doors and body-in-white components. For high production rates, high-pressure die casting (HPDC) is the technique usually employed. The difficulties associated with casting long-solidification-range alloys have been addressed by modifying the alloy composition, and automakers have successfully used vacuum die casting to prepare components from these alloys [1,2,3,4]. Table 1 lists common aluminum foundry and die-cast alloys.

Gowri and Samuel [5] studied the effect of the solidification rate (0.3–20 °C/s) on the solidification behavior of Al–Si–Mg cast alloys and concluded that the dendrite arm spacing (DAS) can be related to the solidification rate (T) using the following expression:

DAS = A(T)^−n^ (1)

where A is a constant and n = 0.33. In another study [6], the same authors investigated the relationship between the alloying elements added to 380 alloys, mainly 3.22 to 4.09% of Cu, 1.01 to 1.70% Fe, 0.06 to 0.50% Mg, 1.69 to 3.00% Zn, and 0.16 to 0.46% Mn. The alloys were solidified at a slow rate (~0.4 °C/s) leading to the precipitation of multiple phases. Samuel et al. [7] extended the study of Mg addition (0.06 wt% in the base alloy, 0.33 and 0.5 wt%) on the hardness of 380 alloys following T6 treatment. The authors observed that increasing the Mg content beyond 0.3% did not yield a further noticeable increase in the alloy hardness. On the other hand [8], porosity was reported to increase from metallographic examination of the fractured test bars. Anilchandra et al. [9] concluded that the improvement in the alloy tensile strength of castings produced by pressure die casting is superior to those made using gravity die casting, and is mainly attributed to minimum defects and imperfections.

The application of the high-pressure die-casting process [10,11] has resulted in significant achievements in the production of (Al–Si) based alloys that are processed using gravity die casting. It is recommended that the injection pressure, as well as the die temperature (and hence the solidification rate), are the main parameters to be considered in this respect. In addition [12,13], the iron precipitates in two distinct primary forms, namely, hexahedron and spherical. Another property to be examined is the fatigue life of pressure die-cast components. In general [14], the applied load must be distributed uniformly over the sample gage length to avoid buckling. Garb et al. [15] emphasized the role of porosity distribution and the location of maximum pore size. Large samples need special care due to design imperfections that cannot be eliminated entirely during sample machining since these imperfections result from the heterogeneous distribution of the applied stresses [16]. According to Isakov et al. [17], who used the rotating–bending fatigue technique for their tests, the scattering intensity of fatigue data is mainly related to the number of crack initiation and propagation sites. Murakami [18] added that another factor to consider would be the inclusion size at the crack initiation point. From the work of Wang et al. [19], the crack configuration is directly linked to the Si content of the alloy in the sense that a high Si content would lead to a tortuous crack propagation path against branched crack propagation in the case of low-Si containing alloys.

According to Samantha et al. [20] and Kolahdooz et al. [21], the application of friction stir processing to 380 alloys would lead to the breaking down of both aluminum dendrites and acicular Si particles which are much finer than those obtained using HPDC. Also, the interparticle distance is reduced by about 50%, resulting in significant improvements in the alloy hardness due to low porosity and microstructural homogeneity. The microstructure and properties of V-treated 380 alloy processed by the rheo-squeeze casting technique were investigated by Lin et al. [16]. The results show that by increasing the V concentration from 0% to 1.05%, the average length and volume fraction of the β-Al_5_FeSi phase is reduced to 30 µm and 1.44%, respectively. When the melt is solidified under high pressure, the solute diffusion coefficient is decreased, whereas the liquidus temperature and solid solubility of the solute element are both increased [22,23,24,25]. This process leads to the precipitation of different phases coupled with marked grain refining which may be attributed to the precipitation of fine Si_2_V phase particles. Another parameter to be considered is the addition of Mg. The work of Istrate et.al [26] reveals the interaction of Mg with other elements such as Zr, resulting in uniform grains. Salahshoor and Guo [27] confirmed that the interaction between Mg and Ca (which exists as a tramp element in commercial alloys) results in the formation of a Mg_2_Ca compound/intermetallic phase that precipitates on the grain boundaries, restricting their growth.

The present study was part of a program established at the Université du Québec à Chicoutimi in 1994 in collaboration with General Motors, USA, and Corporativo Nemak, Mexico. The program was aimed at investigating different parameters that would enhance the performance of 380 alloy due to its wide use in automotive industries. Although the previous studies demonstrated the role of alloying elements such as Fe, Mg, Cu, and Zn on the microstructure and tensile properties of A380.1 alloy, the tensile bars were produced employing the gravity die casting technique (maximum solidification rate of about 8 °C/s) which does not represent actual industrial needs. Thus, the work was extended to include the application of a low-pressure die-casting technique (solidification rate approximately 800 °C/s). The role of increasing the Mg content, as well as aging conditions were also analyzed.

## 2. Experimental Procedure

### 2.1. Preparation of Tensile and Fatigue Samples

**Figure 1:**
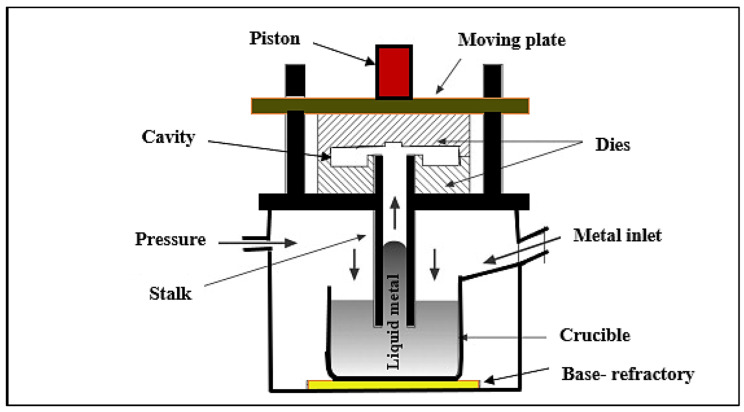
Schematic of the die-casting process.

Two main alloys were used: an experimental alloy containing Al-9.5%Si-3.5%Cu, and an industrial alloy coded A380.1 alloy (see Table 2). The A380.1 alloy was melted in an electrical resistance furnace using a SiC crucible of 60 kg capacity. The alloy was melted at 750 °C ± 5 °C and degassed for 30 min using pure Ar gas that was introduced into the molten bath through a graphite impeller rotating at 130 rpm. The Mg concentration in the A380.1 alloy was increased to 0.33 wt% (similar to that for an A356 alloy), then to 0.55 wt% (corresponding to the Mg level in an A357 alloy). In order to minimize Mg oxidation, Be was added in the amount of 300–400 ppm using an Al-5%Be master alloy (similar to the technique used by Alcoa). The melting was carried out using a special ventilation setup. The molten A380.1 alloy was poured into a preheated metallic mold (triangle web beam bar-type) coated with boron nitride. The triangular-shaped ingot bars produced from this mold were used for die casting (ingots weighed approximately 3 kg each). Table 3 shows the final compositions of the three alloys used, while Figure 1 shows a schematic diagram of the die-casting process.

**Figure 2:**
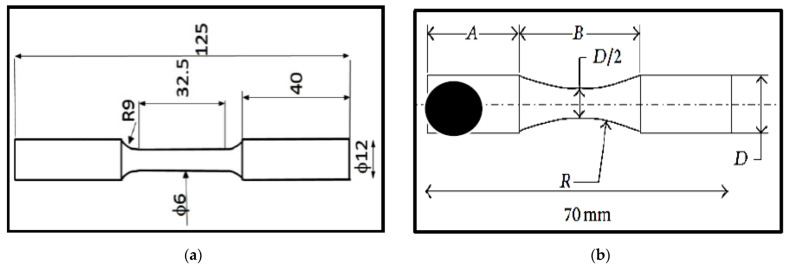
(**a**) Tensile sample—ASTM E-8M, (**b**) Fatigue sample—ASTM E 466—all dimensions are in mm.

All new ingots were pressurized at CANMET (Ottawa, ON, Canada) at 1 bar. The dies were not preheated to obtain the highest possible solidification rate, instead, the molten metal was kept at 750 °C and no degassing was applied. Figure 2 shows the shape and dimensions of the samples used for tensile and fatigue testing. All samples were solution heat treated in an air-forced furnace at 495 °C (±2 °C) for 8 h, followed by quenching in warm water at 60 °C. Quenched samples were thereafter aged at 155 °C (T6) or 220 °C (T7) for times of up to 25 h, followed by air-cooling.

**Figure 3:**
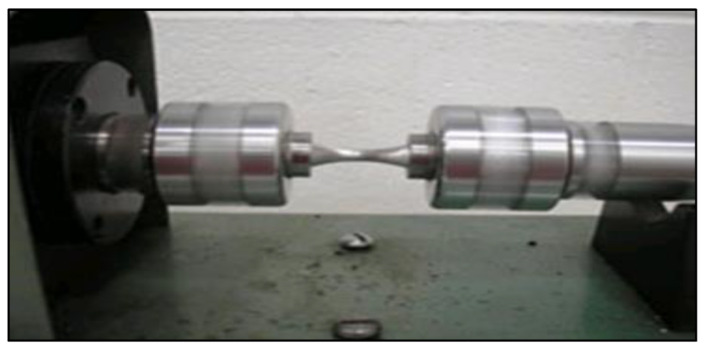
Bench-type rotating–bending fatigue testing apparatus.

Samples for tensile testing were pulled to fracture using an MTS Servohydraulic machine at a strain rate of 4 × 10^−4^/s. A one-inch extensometer (2.54 cm) was attached to the gage length to record the deformation. The data acquisition system records the ultimate tensile strength (UTS), yield strength (YS), or more precisely a 0.2% offset YS, and the percent elongation (%El). For each case/condition, five tensile bars were tested and the average was reported. Figure 3 demonstrates the bench-type machine used for the rotating–bending fatigue test in the present study.

### 2.2. Thermal Analysis

**Figure 4:**
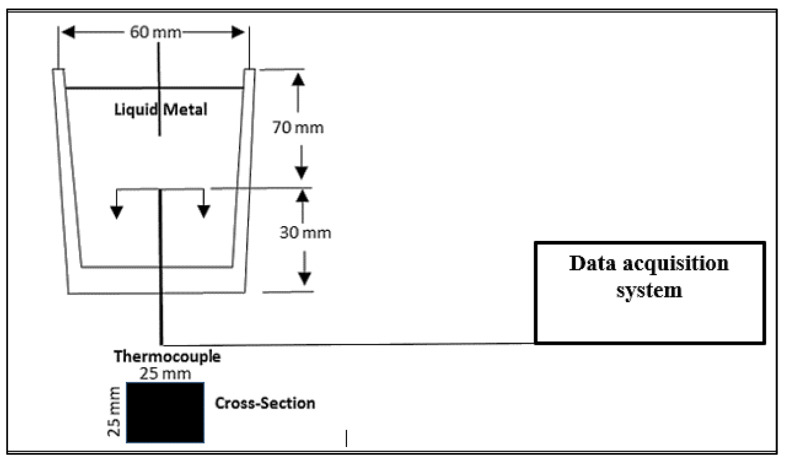
Schematic diagram of the thermal analysis setup used to produce samples solidified at a close-to-equilibrium solidification rate, for the analysis of the precipitated phases.

To obtain the solidification curves of the four studied alloys at a solidification rate near equilibrium (~0.35 °C/s), approximately 800 g of each alloy was melted in an electrical resistance furnace using a 2 kg capacity SiC crucible. A thermocouple (Type-K chromel-alumel) was inserted from the center of the bottom of the crucible up to about half of its height. The thermocouple was attached to a data acquisition system that recorded the temperature data every 0.1 s. A schematic diagram of the setup is provided in Figure 4.

### 2.3. Metallography and Fractography

The samples for grain size measurements were sectioned from the gage length of the tensile sample (as-cast condition), and from the cylindrical graphite mold casting, sectioned such that the top surface contained the thermocouple tip (thermal analysis). The samples were polished and etched in a reagent composed of 66% HN0_3_, 33% HCl, and 1% HF. The samples for optical metallography were sectioned from the same positions and polished prior to examination. No chemical etching was applied. An Hitachi SU-8000 field-emission scanning electron microscope (FESEM) (Hitachi High-Technologies Corporation, Tokyo, Japan) equipped with detectors for energy dispersive X-ray (EDX) and wavelength dispersive spectroscopic (WDS) analyses was used for phase identification and to characterize the fracture behavior of both tensile- and fatigue-tested samples.

## 3. Results and Discussion

### 3.1. Thermal Analysis

**Figure 5:**
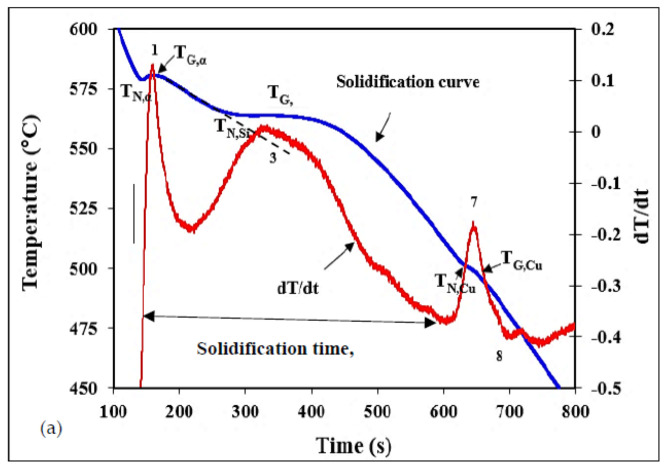

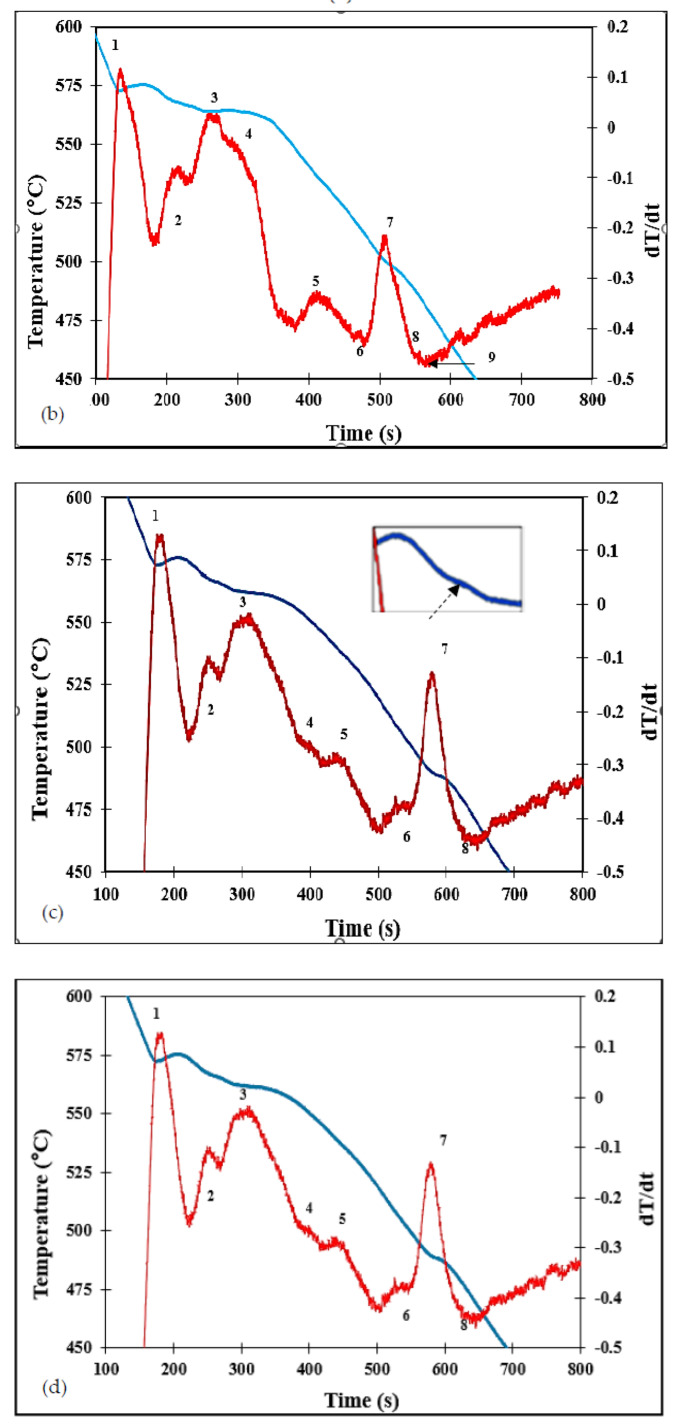
Solidification curves and their first derivatives from samples solidified at the rate of ~0.35 C/s: (**a**) experimental alloy, (**b**) alloy A, (**c**) alloy B, and (**d**) alloy C.

**Figure 6:**
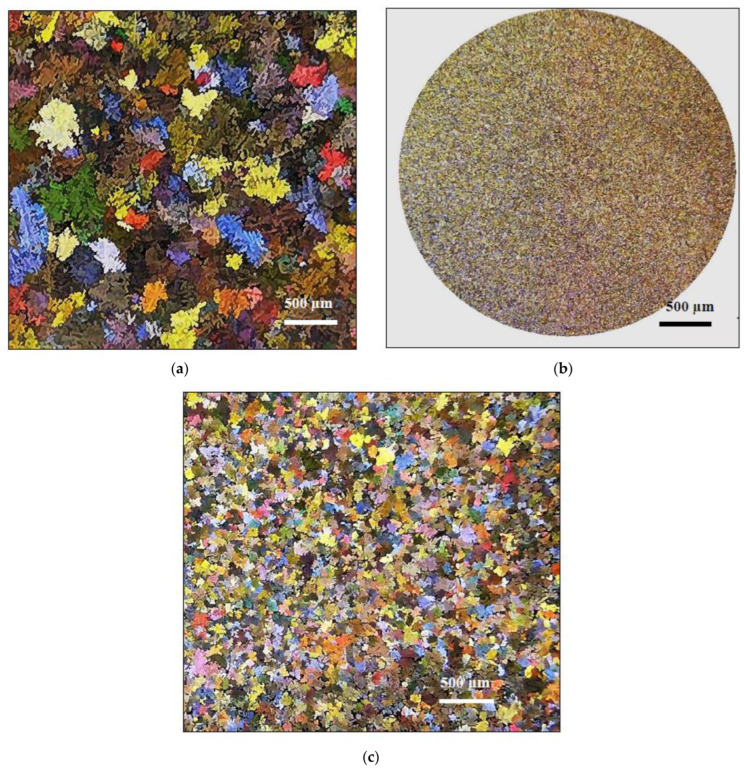
Macrostructure showing grain size in samples sectioned from (**a**) thermal analysis casting, (**b**) die casting, and (**c**) thermal analysis sample grain refined with 0.15% Ti (added as Al–5%Ti–1%B master alloy to alloy A).

Figure 5a illustrates the solidification parameters of the experimental alloys and undercooling associated with the three main reactions (see Table 4). In the present work, solidification time will be used to compare the solidification rate for all four studied alloys rather than the slope of the curve in the mushy zone (broken line). In a simple alloy, using the slope normally gives close values to those reported in the dendritic arm spacing as a function of solidification rates, as developed by Grant [28]. However, in complex alloys containing several alloying elements, and hence, many reactions (see Figure 5d, for example), it is difficult to determine the straight line with high accuracy due to its irregular shape (see dashed arrow in the inset of Figure 6c). Another observation that can be made from Figure 5a is that the undercooling (T_G_-T_N_) associated with the development of the α-Al network is more explicit than those observed for the (Al–Si) and (Al–Al_2_Cu) eutectic reactions. The importance of the ∆T_α_ undercooling is that it defines the degree of the alloy grain refining [29,30].

It has been reported that the addition of a high percentage of Fe to die-cast alloys prevents the soldering of the casting with the steel die. In this case, a coating is not needed [16,31,32,33,34] which is associated with the peak marked 2 in Figure 5b–d. According to the work carried out by Ji et al. [35] and Buchanan et al. [36] on the effect of Mg content on the precipitation of the Q–Al_8_Mg_3_FeSi_6_ phase, when the Mg content in a 319 alloy is less than 0.3 wt%, the Q-phase precipitates following the formation of eutectic Al–Al_2_Cu. At higher Mg concentrations, the Q-phase precipitates before as well as after the eutectic reaction, as seen by the two peaks in Figure 5d corresponding to alloy C containing 0.55 wt% of Mg. The precipitation/reaction sequence was determined in the light of the description provided in ref [37].

### 3.2. Macrostructure and Microstructure

**Figure 7:**
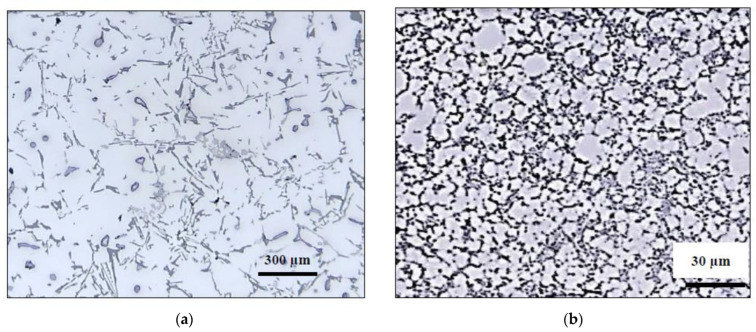

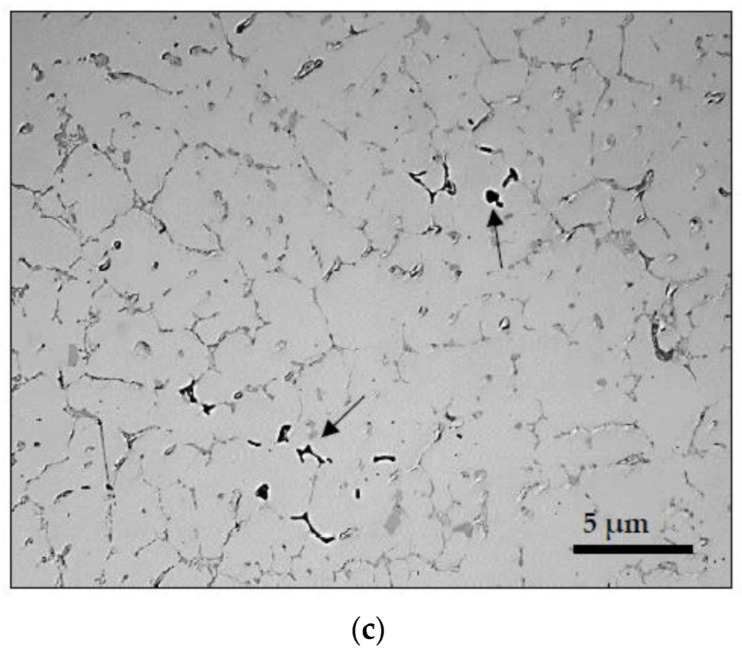
Optical microstructure obtained from samples taken from (**a**) thermal analysis casting, (**b**) die-cast alloy A, and (**c**) porosity observed in (**b**)-black arrows.

Figure 6 reveals the grain structure of samples taken from castings obtained from thermal analysis and die-casting techniques. The grain size in Figure 6a is in the range of 400–600 µm, whereas that sectioned from the pressurized tensile bar is about 3–5 µm, Figure 6b, indicating the effectiveness of solidification as a strong grain refiner compared to that obtained from the grain size of the thermal analysis samples which were refined using TiBor—approximately 100–150 µm—as shown in Figure 6c. It is inferred from Figure 7 that the average dendrite arm spacing of the thermal analysis sample is in the range of 100–150 µm compared to ~2–5 µm measured from the die-cast sample. According to Grant’s chart, the corresponding solidification rates are 0.35 °C/s (which is more or less close to that obtained from the solidification curve in the case of the experimental alloy), and 800 °C/s, respectively.

Another observation noted from Figure 7 is that the average eutectic Si particle length is about 200–300 µm, compared to that produced by applying a solidification rate of ~800 °C/s, approximately 1 µm—Figure 7b. Figure 7c depicts the presence of microporosity (less than 1 µm) dispersed throughout the microstructure. In addition, the dendrite arm spacings in Figure 7a are almost 60 times higher than those in Figure 7b.

**Figure 8:**
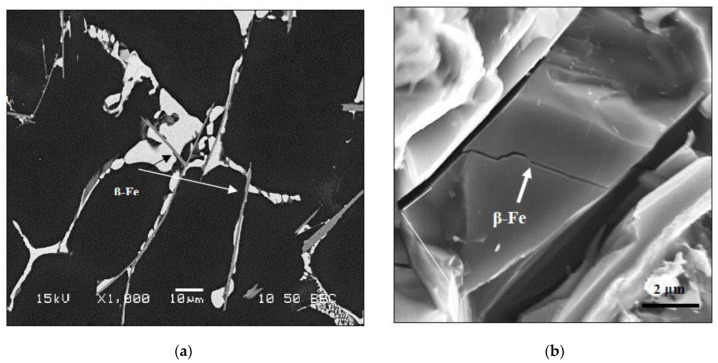

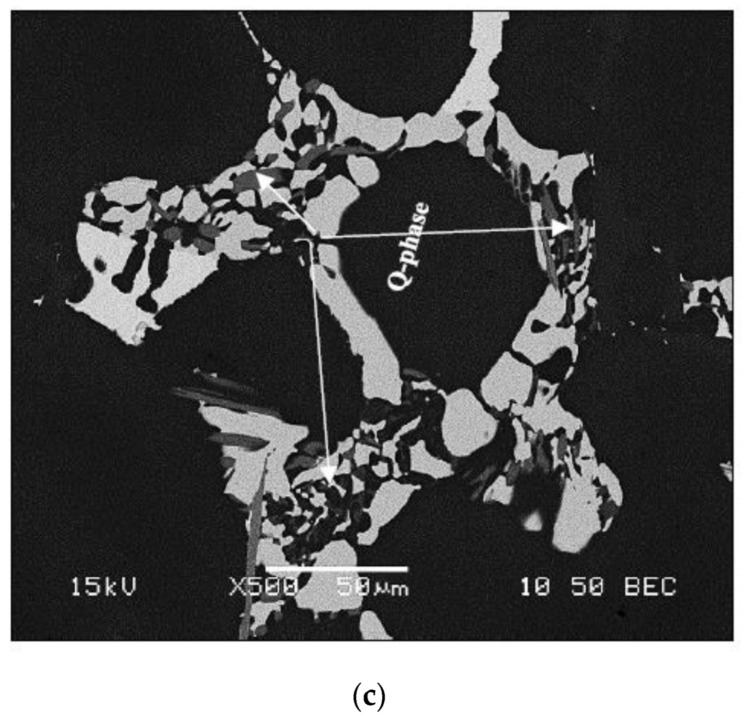
Backscattered electron images viewed from alloy A (0.35 °C/s) in the as-cast condition, showing (**a**) β-Fe phase platelets existing with massive Al_2_Cu particles; (**b**) fracture of a β-platelet; (**c**) particles of Q-phase growing out of the (Al-Al_2_Cu) eutectic network.

In order to understand the role of precipitated intermetallics, samples prepared from alloy A solidified at a slow rate were examined using the FESEM technique, as depicted in Figure 8. Figure 8a highlights the presence of β-Al_5_FeSi platelets co-existing with massive areas of Al_2_Cu phase particles. Figure 8b demonstrates the fracture surface of alloy A in the as-cast condition (from a tensile-tested bar sample—solidification rate approximately 8 °C/s), revealing the fracture of a β-platelet (note the constraint on the crack within the brittle β-platelet—arrowed). In another view illustrated in Figure 8c, small particles of Q-phase are seen growing out of the (Al–Al_2_Cu) eutectic network. As noted in Table 3, alloy A contains ~0.06 wt% Mg [38].

**Figure 9:**
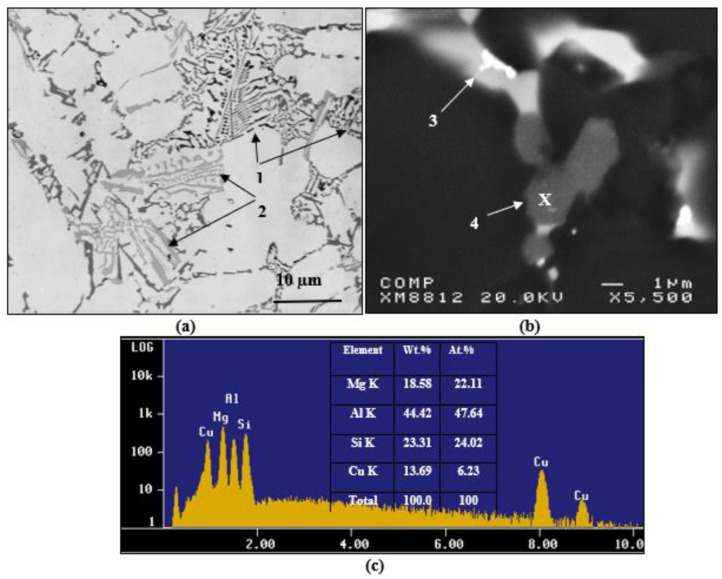
(**a**) Optical microstructure sectioned from alloy C (0.35 °C/s) showing 1—Mg_2_Si, and 2—Π-Fe phase particles, (**b**) backscattered electron image showing 3—Al_2_Cu massive particle, and 4—Q-phase particles, and (**c**) EDS spectrum corresponding to the area marked X in (**b**).

**Figure 10:**
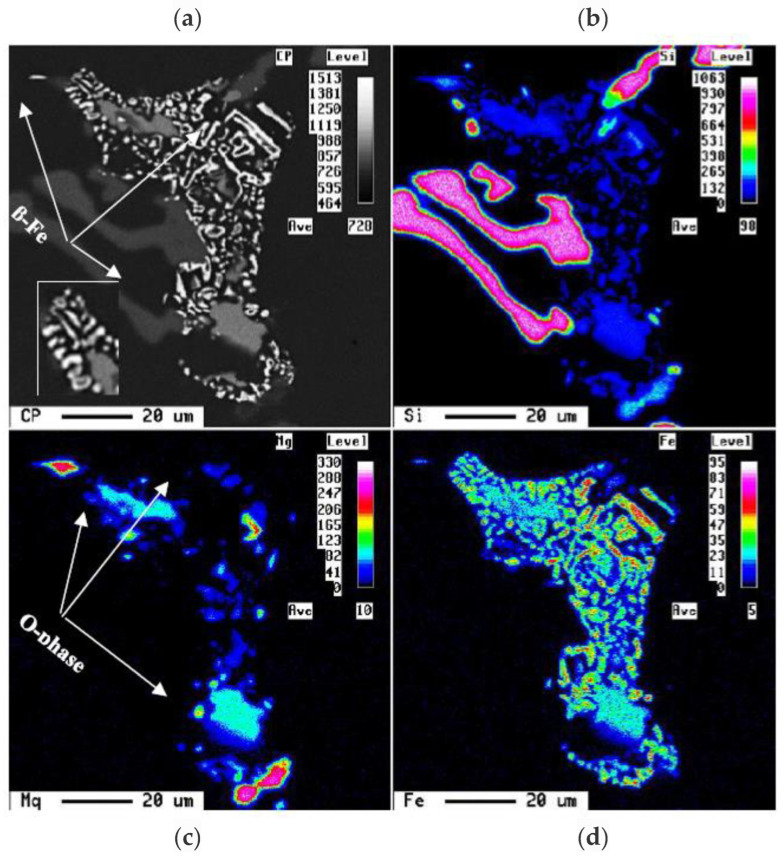
(**a**) Backscattered electron image sectioned from an alloy C sample (0.35 °C/s), and corresponding X-ray maps of (**b**) Si, (**c**) Mg, and (**d**) Fe elements in (**a**). The inset in (**a**) shows the decomposition of π-Fe to β-Fe (white arrows) following solution heat treatment [39].

Increasing the Mg content in alloy C coupled with a slow solidification rate resulted in the precipitation of a large volume fraction of both π-Fe and Q–Al_8_Mg_3_FeSi_6_ phases [39], followed by the precipitation of Mg_2_Si particles, as shown in Figure 9a. Figure 9b reveals the precipitation of Q–Al_5_Cu_2_Mg_8_Si_6_ [38] in the form of large particles located at the edges of the Al_2_Cu phase particles explaining the explicit peak observed in Figure 6d, reaction #6 in Table 4, and confirmed by the EDS spectrum exhibited in Figure 9c corresponding to the area marked X in Figure 9b. According to El-Sharkawi [39], during solution heat treatment at 495 °C for 8 h, the π-phase may dissolve, leading to the formation of fine β-Fe platelets, i.e., a reversible reaction, as depicted in Figure 10.

**Figure 11:**
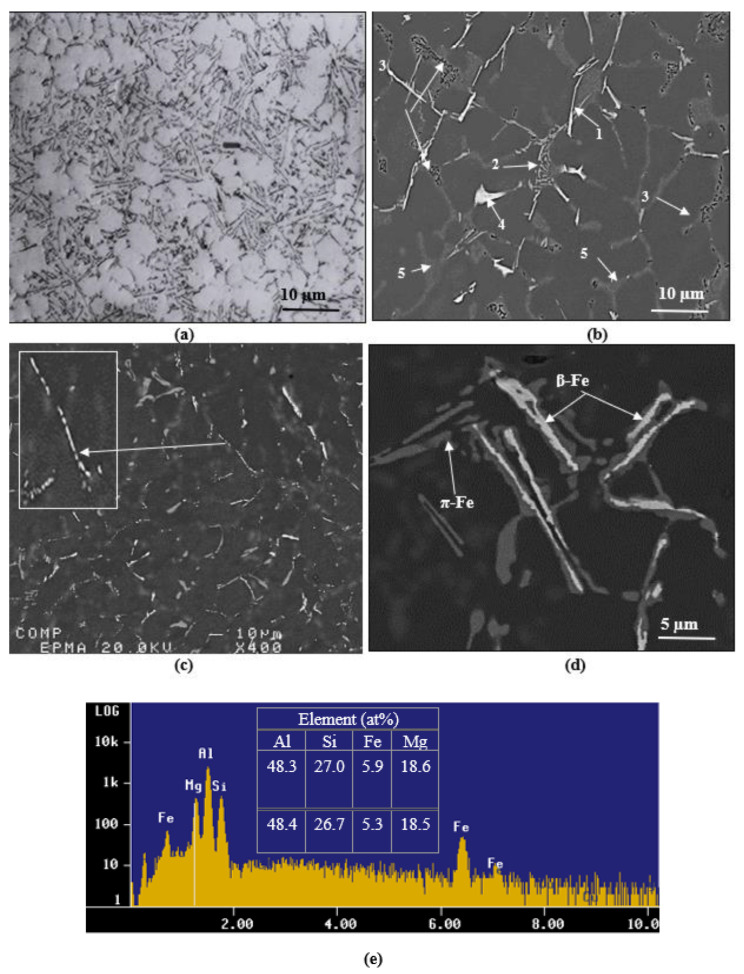
Microstructures of alloy C (800 °C/s) in the: (**a**,**b**) as-cast, and (**c**–**e**) solution heat-treated conditions.

Figure 11 presents a series of microstructures taken from alloy C solidified at ~800 °C/s in the as-cast and solution heat-treated conditions. Figure 11a reveals the fineness of the β-Fe platelets (maximum length < 10 µm). However, other phases are difficult to be seen. The backscattered electron image in Figure 11b more clearly exhibits the size and distribution of the β-Fe platelets (marked 1), π-Fe (marked 2), a large number of fine Mg_2_Si phase particles (marked 3), and some Al_2_Cu phase particles (marked 4). Due to the low atomic number of Si (14) compared to that of the Al matrix (13), the eutectic Si particles (marked 5) appear very faint. The solutionizing treatment of alloy C mainly resulted in the fragmentation of the β-Fe platelets, as illustrated in Figure 11c (see inset), as well as the complete dissolution of both the Mg_2_Si and Al_2_Cu phases. A high-magnification image of part of the area shown in Figure 11b and presented in Figure 11d reveals the transformation of the β-Fe platelets to the π-Fe phase, leading to severe irregularities on the surface of the β-Fe platelets, as confirmed by the associated EDS spectrum shown in Figure 11e.

### 3.3. Tensile Properties and Fractography

**Figure 12:**
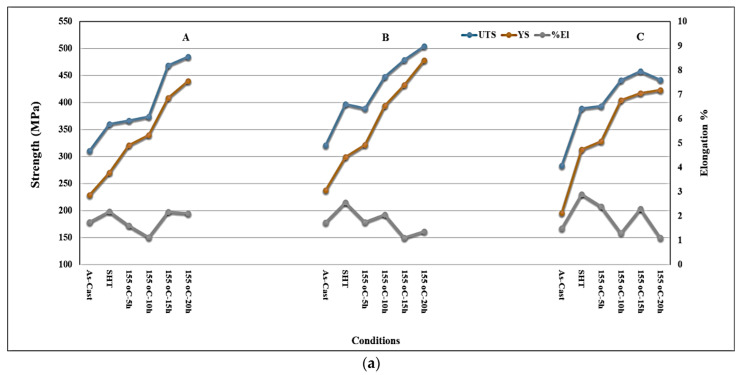

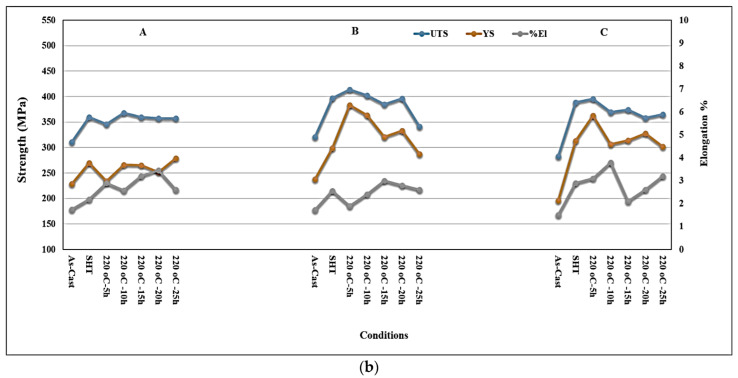
Variation in the alloy tensile properties as a function of the heat treatment condition: (**a**) aging temperature of 155 °C, and (**b**) aging temperature of 220 °C.

In this section, only die-cast alloys will be analyzed. Figure 12 displays the variation in tensile properties of alloys A, B, and C as a function of the heat treatment conditions applied. As can be observed from Figure 12a, there is a marginal increase in both UTS and YS values when the Mg content was increased to 0.33%, which may be attributed to the significance of the solidification rate at this stage over hardening caused by the increase in the Mg concentration. However, the effect of the solidification rate showed a negative tendency when the Mg content was increased to 0.5 wt% due to the formation of a large volume fraction of insoluble intermetallics, in particular, the π-Fe and Q-phases (see Figure 9). From the work of Andrade et al. [40], it was found that the aging curves of high Mg-containing 319 alloy were characterized by the presence of several peaks and values when the alloys were aged in the range 155 °C–220 °C. These peaks were not at the same level of strength as the aging time increased, and occurred due to the simultaneous precipitation of certain hardening-phase particles and a coarsening of the precipitated particles of preceding phases. Thus, it is difficult to draw a direct relationship between UTS and % Elongation. Similar observations can be made from Figure 12 at a lower magnitude compared to those reported by Garza Elizondo et al. [38]. In addition, regardless of the aging temperature, the tensile strength parameters of the 0.5% Mg-containing alloy, i.e., alloy C, are somewhat lower than those reported for alloy B under similar aging conditions.

**Figure 13:**
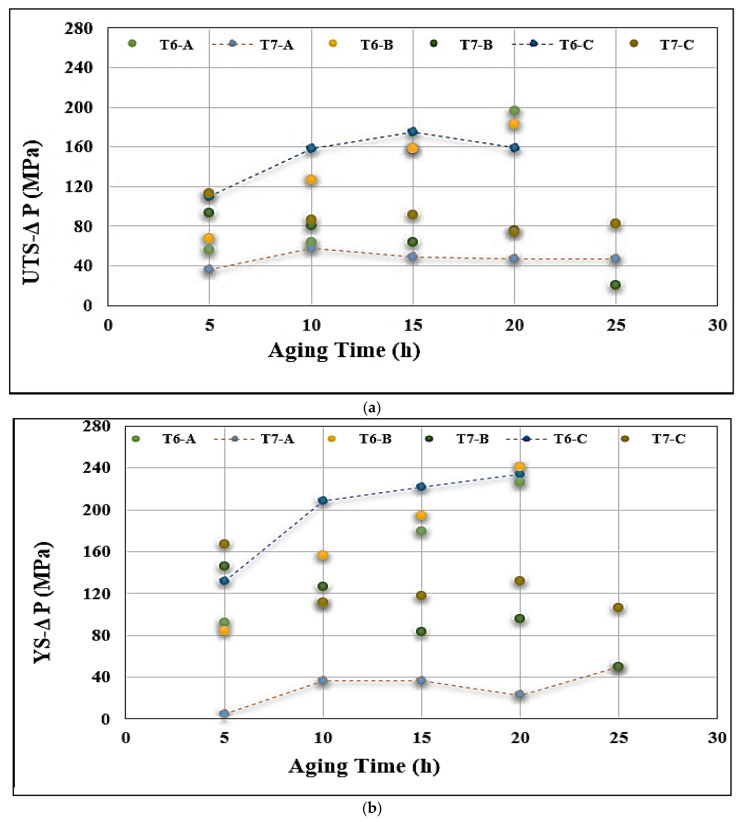

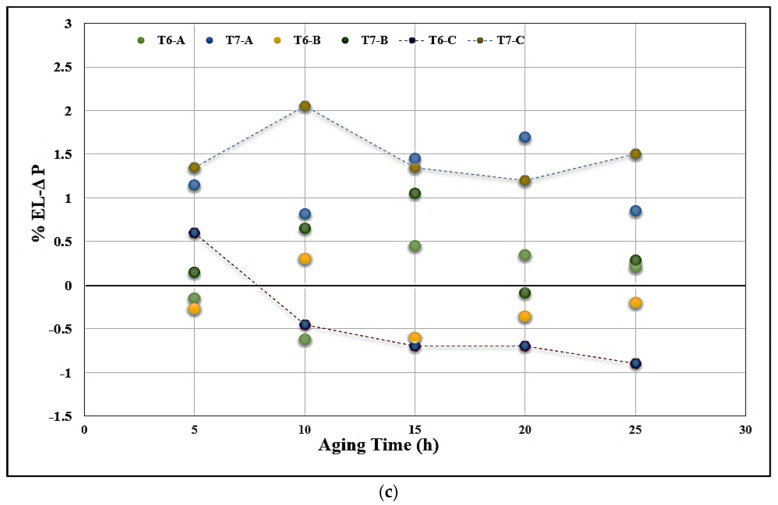
Contribution of Mg content and aging to the tensile properties of alloy A in the cast condition: (**a**) UTS, (**b**) YS, and (**c**) % El.

Table 5 lists the tensile parameters of alloy A in the as-cast condition. These values will be taken to represent the reference level for the calculation of the contribution (∆P) made by the increase in Mg as well as the aging treatments toward the tensile properties, as depicted in Figure 13. Analysis of the data presented in Figure 13 shows that the T6 tempering (aging at 155 °C) of alloy C reaches its maximum improvement in UTS after 15 h (~175 MPa), whereas a minimum contribution was obtained when alloy A was aged at T7 (220 °C) for 15 h—Figure 13a. It is clear from Figure 13b that the contribution of these two conditions to the as-cast alloy A is more significant than their influence on the alloy UTS levels, e.g., 226 and 22 MPa, respectively. Figure 13c illustrates the variation in ductility of as-cast alloy A revealing that only the heat treatment in the T7 temper would enhance its % elongation ~by 2% (i.e., 115% of the as-cast value for alloy A), whereas for the T6-tempered alloys, the ductility decreases by more or less the same value which is about 60% of the percent elongation value of alloy A in the as-cast condition.

The contribution ∆P is calculated as:

∆P = P_x_ − P_A(as cast)_ (2)

where P represents the tensile property, and P_A_ and P_X_ are the property values in the as-cast condition and the alloy condition under consideration, respectively.

**Figure 14:**
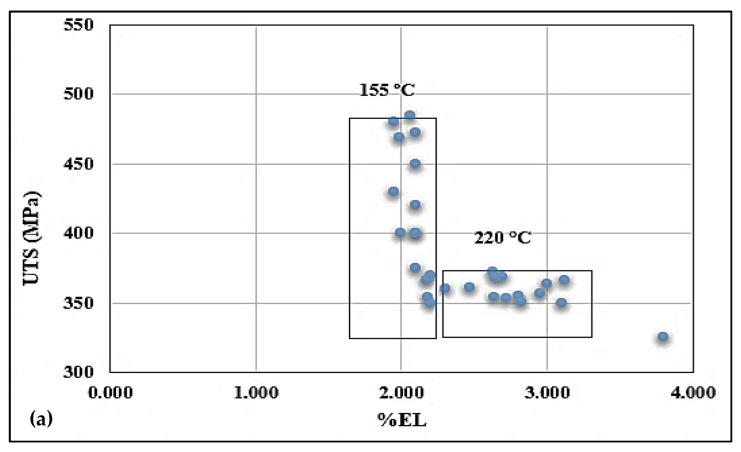

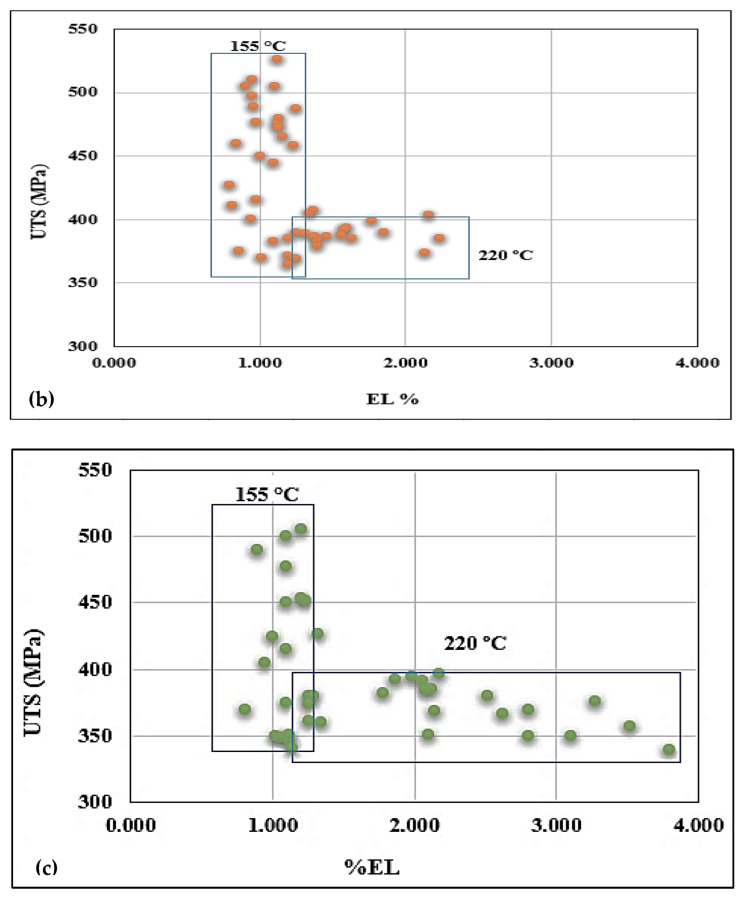
Plots of UTS vs. % Elongation for (**a**) alloy A, (**b**) alloy B, and (**c**) alloy C.

The work of Garza Elizondo et al. [38] on 354 alloys (containing 1.6% Cu and 0.58% Mg) revealed a linear relationship between UTS and ductility. In the present study, as Figure 14 shows, an L-type relationship is observed for each alloy divided into two blocks: a vertical block containing values corresponding to 155 °C aging temperature, and a horizontal block of values corresponding to samples aged at 220 °C. The difference between the three alloys lies in the positioning of the blocks with respect to both the X and Y axes. Figure 14a shows that most of the points are skewed away from the UTS axis compared to the points presented in Figure 14b, and c. In addition, Figure 14c reveals that the points are widely spread over a longer range of percent elongation and a wider range of UTS values. In other words, alloy C is similar to alloy B, but on a larger scale of distribution.

In 1980, Drouzy et al. [41] proposed what they called the quality index (*Q*) to classify the quality of aluminum castings (Al–7% Si–0.3% Mg 356 alloy):

(3) $$Q\;=\;\;\sigma_{uts}\;+\;d\;\log\;(E_{f})$$

where *Q* is the quality index in MPa; $\sigma_{uts}\;$ represents the ultimate tensile strength in MPa; *E_f_ is* the percentage elongation to fracture; and *d* is a material constant equal to 150 MPa. The probable yield strength (σ*_P_*_(*YS)*_) for the same alloy may be expressed as:

(4) $$\sigma_{P(YS)}\;=\;\;\;a\;\sigma_{UTS}\;-\;b\;\log\;(E_{f})\;+\;c$$

where the coefficients *a*, *b*, and *c* for Al–7Si–Mg were used as 1, 60, and −13, respectively, in MPa.

**Figure 15:**
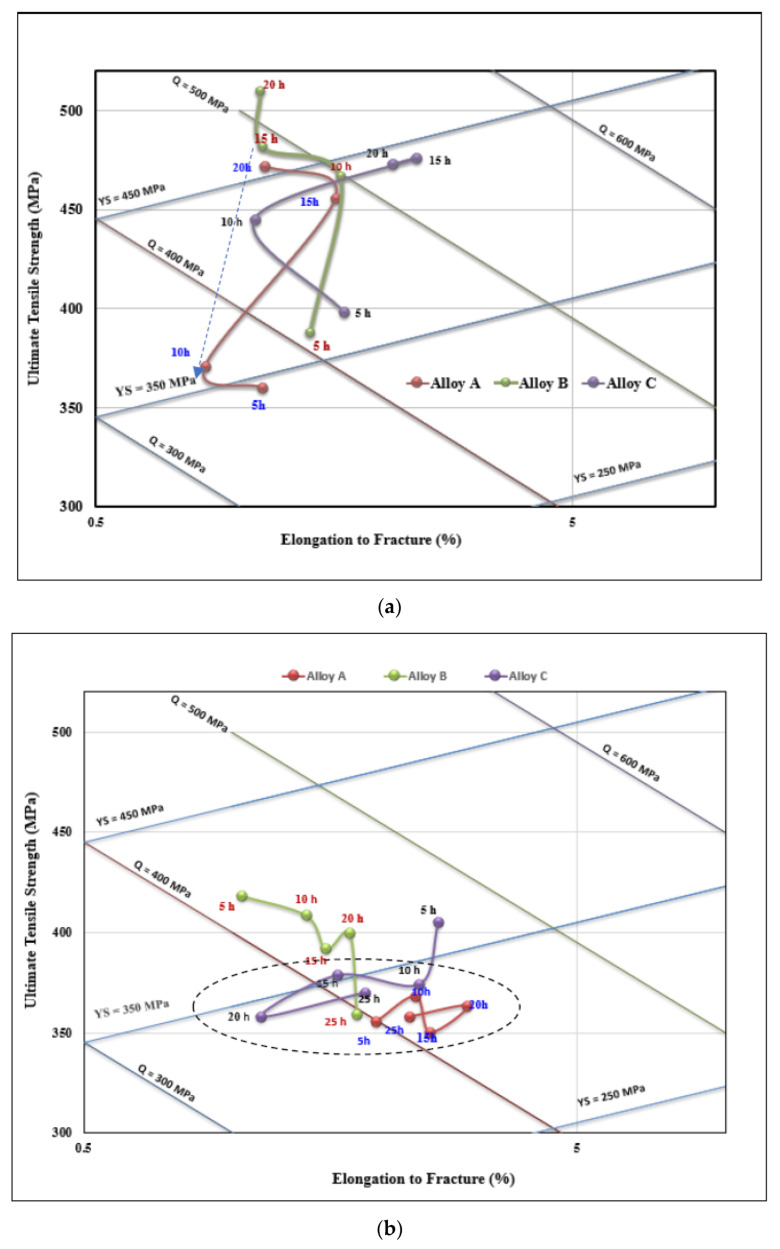
Q-charts calculated using Equations (3) and (4) for (**a**) 155 °C, and (**b**) 220 °C aging temperatures.

Figure 15 depicts the quality index charts for alloys A, B, and C based on Equations (3) and (4). In Figure 15a, samples were aged at 155 °C, showing each plot in the form of a curve where the nose of the curves lie in the UTS range of 470 MPa (alloy B—15 h aging)—360 MPa (alloy A—10 h aging), see the blue dashed arrow, and elongation to rupture ranging between 0.8% and 2.6%. Considering the *Q*-levels, the alloys are falling in the range of 540 MPa (alloy C—15 h aging) and 460 MPa (alloy A—10 h aging). Unlike aging at 155 °C, most of the points of the samples aged at 220 °C fall within a narrow area, as denoted by the dashed circle in Figure 15b. The observed multiple peaks are related to the scatter in UTS values observed in Figure 12.

**Figure 16:**
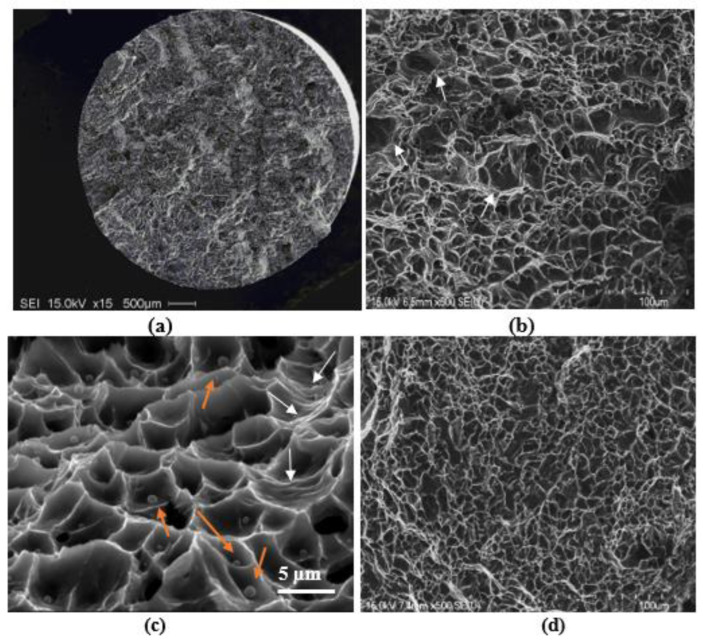
SEM of the fracture surface of alloy A under different conditions: (**a**) as-cast-general view, (**b**) high-magnification view of the central portion, (**c**) following solution heat treatment, and (**d**) in the T6 condition (20 h at 155 °C corresponding to maximum UTS level). Note the presence of spherical Si particles at the bottom of the dimples in (**c**) orange arrows, and slip bands, white arrows.

Figure 16 shows some examples of the fracture behavior of alloy A. A general view is presented in Figure 16a, revealing no visible defects either on the outer sample surface or on the fracture surface itself. A high-magnification micrograph of Figure 16a exhibits a well-defined fine dimple structure (less than 10 µm) mixed with cleavage areas (white arrows), as displayed in Figure 16b. Solutionizing at 495 °C for a period of 8 h brought about a significant improvement in the uniformity of the dimple structure. In this case, the dimples appear to be much larger and deeper than those shown in Figure 16b.

In addition, slip bands are frequently observed covering the inner surfaces of the dimples—see white arrows in Figure 16c. With the marked increase in the alloy strength following aging at 155 °C for 20 h, i.e., 360 MPa (SHT) to 470 MPa (T6), the fracture displayed a fine dimple structure similar to that observed in Figure 16a with no noticeable cleavage areas, as demonstrated in Figure 16d.

### 3.4. Fatigue Behavior

In this section, the results of low fatigue cycles of the alloys using the rotating–bending technique will be discussed. The fatigue data are normally plotted in the form of S–N diagrams. The number of cycles N is shown on the X-axis and the stress S on the Y-axis. In contrast to steels, for the aluminum alloys, there is no horizontal asymptote. Therefore, failure is inevitable regardless of the stress imposed, as long as the number of cycles is sufficient with an endurance limit defined as the breaking stress for 5 × 10^8^ cycles [42]. Loads that cannot produce failure in a single application may eventually cause the failure of a part if their application is repeated a sufficient number of times. It is therefore possible to study the proportionality of this number of cycles with the applied stress [43]. In all cases, the applied stress was a percentage of the yield stress.

**Figure 17:**
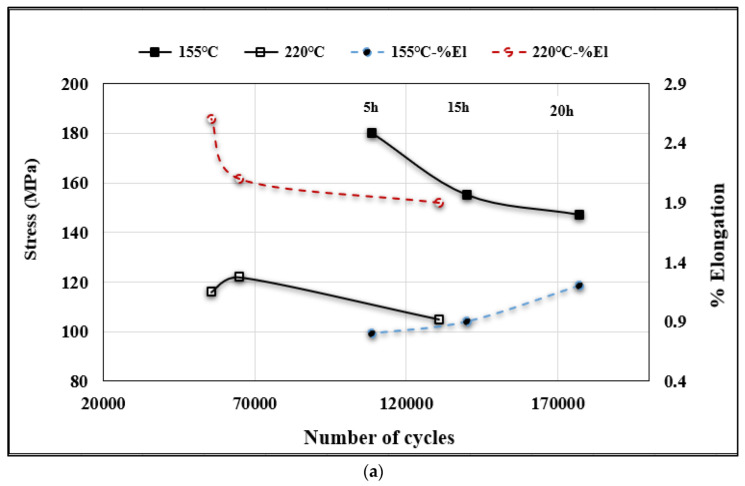

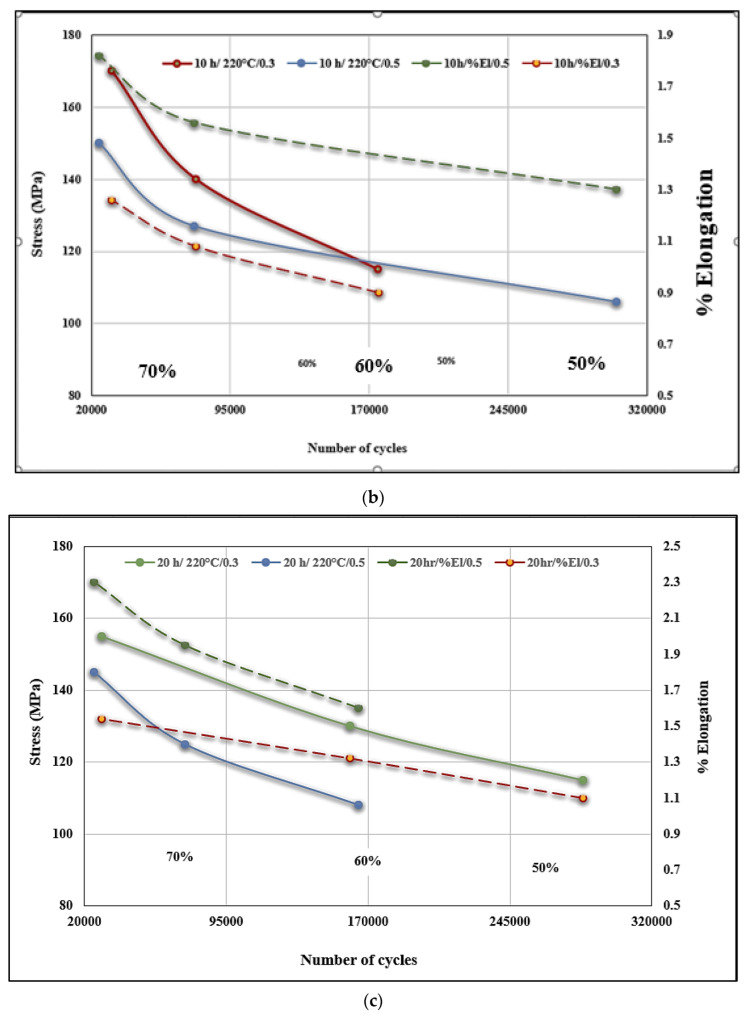
S–N plots for (**a**) alloy A aged at 155 °C and 220 °C for different times; (**b**) alloys B and C aged for 10 h/220 °C; and (**c**) alloys B and C aged for 20 h/220 °C.

Figure 17 depicts a series of S–N curves displaying the combined effect of Mg content and aging conditions. As for alloy A, only 50% of the yield stress was applied and the results are shown in Figure 17a. As may be seen, aging at 155 °C achieved higher levels of the number of cycles prior to fracture compared to samples treated in the T7 condition. However, for both treatments, the range of N is about 70–75 cycles. In addition, the percentage elongation to fracture vs. N reveals the dependence of the fatigue life on the alloy ductility. The S–N plots in Figure 17b were constructed using 50, 60, and 70% of the alloy yield stress of B and C alloys in the T7 tempered condition (10 h at 220 °C). It is evident that low stress coupled with high ductility would lead to a longer fatigue life compared to the application of higher stresses. It should be mentioned here that the plotted % elongations are also the same in value as the percentage of the alloy total elongation to fracture. Similar observations were noted for alloys aged at 220 °C for 20 h—see Figure 17c.

**Figure 18:**
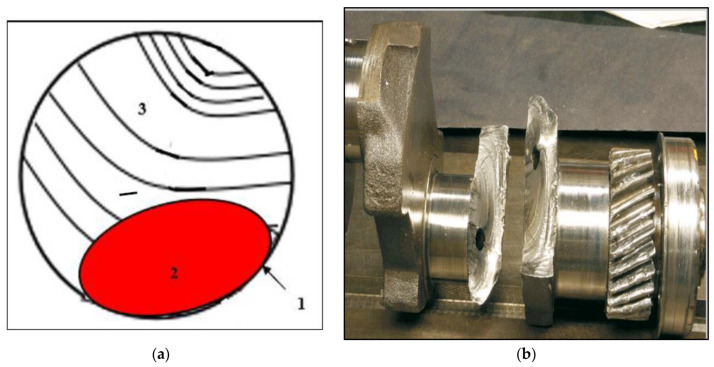
(**a**) The proposed model of fracture by Yang et al. [44], and (**b**) fracture of a steel gearbox.

Yang et al. [44] studied the effect of rotating–bending fatigue on the lifetime of a 7075 alloy in the T6 condition. Their observations show that the fatigue crack initiation takes place at the sample outer surface (marked 1) and propagation (a smooth rubbed surface-marked 2), followed by a rough area in relief indicating final break (marked 3), as illustrated schematically in Figure 18a. However, this type of fracture normally occurs under mechanical fatigue testing caused by alternate compression–tension, an example of which is shown in Figure 18b.

**Figure 19:**
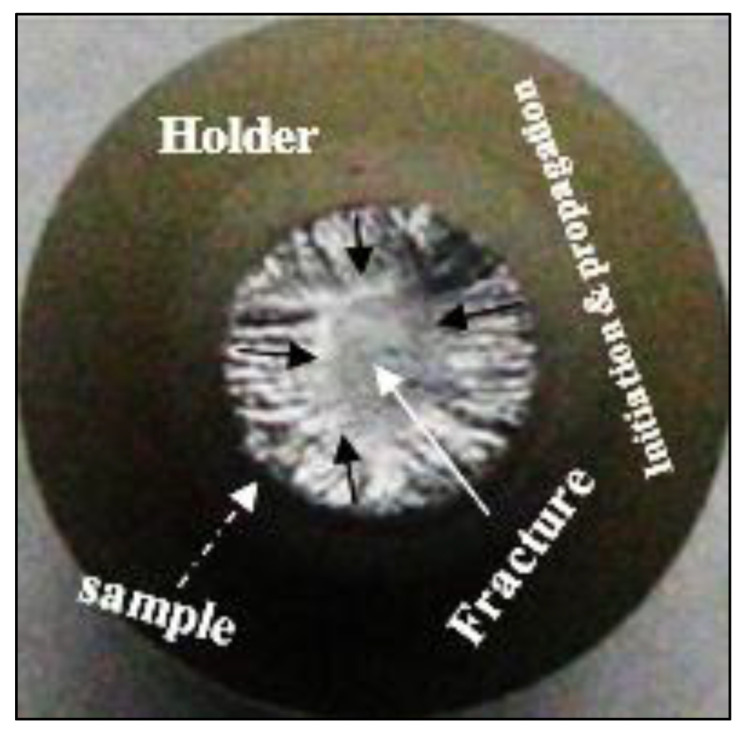
Fracture of fatigue-tested samples in the present work.

Figure 19 demonstrates the fracture mechanism of the present fatigue-tested bars. The crack commenced from the outer surface due to certain defects and propagates radially toward the center of the samples (black arrows); this leads to an increase in the applied stress, ultimately reaching the fracture point—see the white arrow which is in contrast to the model proposed in [44].

**Figure 20:**
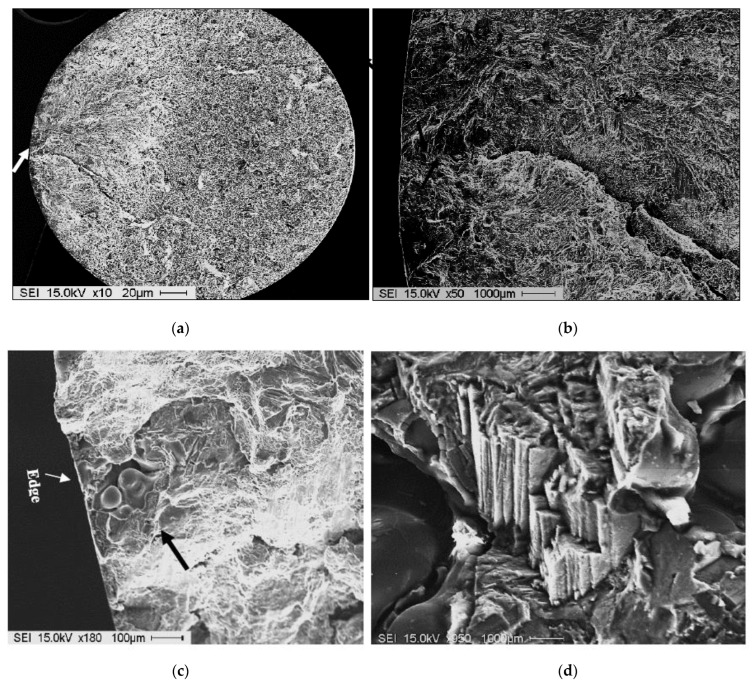

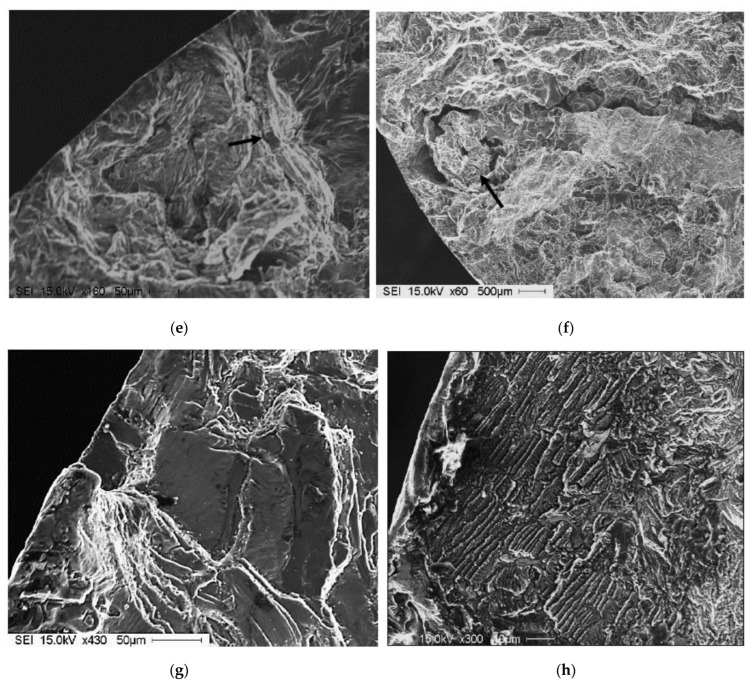
Examples of crack initiation sites: (**a**,**b**) crack, (**c**) porosity, (**d**) slip bands, (**e**,**f**) oxide films and inclusions, (**g**) cleavaged areas, and (**h**) river-line striations.

Figure 20 depicts most of the sources of fractures viewed in the present work. As shown in Figure 20a,b, a long crack was initiated at the sample outer surface and propagated toward the center, as indicated by the dashed arrow in Figure 19. The presence of gas or shrinkage porosity is one of the most deleterious causes of crack initiation, as shown in Figure 20c. In the absence of porosity, slip bands could as well lead to initiating the crack, as seen in Figure 20d. However, they have a lesser effect on fatigue behavior compared to surface porosity. Another important element is the entrapment of oxide films (arrowed in black) and/or inclusions (arrowed in white) in the casting, as shown in Figure 20e, and f, respectively, whereas Figure 20g reveals severe cleavage sites. As mentioned in the experimental section, the molten metal was only degassed before ingots were shipped to the die-casting facilities. River-line striations created by a high number of fatigue cycles (see Figure 17b,c, for example) were frequently seen, as exemplified in Figure 20h.

## 4. Conclusions

Based on the results obtained from the present work, the following conclusions may be drawn:

1. At a high Mg content in 380 alloys, i.e., 0.5%, the Q–Al_5_Mg_8_Si_6_Cu_2_ phase precipitates in the form of large particles at the edges of the Al_2_Cu phase particles instead of growing out of the (Al–Al_2_Cu) eutectic structure. In addition, its precipitation temperature is higher than that of the Al_2_Cu formation temperature.
2. Both Mg_2_Si and π-phase precipitate in a relatively large volume fraction and size.
3. The use of the slope of the solidification curve in the mushy zone for measuring the solidification rate is not accurate due to the presence of several irregularities caused by the precipitation of different phases in this zone. It is suggested that the solidification time should be used instead.
4. Low-pressure die casting using a cold chamber and small samples would result in a solidification rate of the order of about 800 °C/s corresponding to a dendrite arm spacing of 3–5 µm, compared to 85 µm for castings solidified at a slow rate (~0.35 °C/s).
5. Solution heat treatment at 495 °C for 8 h causes the transformation of β-Fe to π-Fe that leads to a thinning of the β-Fe platelets with the appearance of multiple irregularities on the surface of the Fe platelets.
6. Alloys containing 0.5% Mg offer tensile properties somewhat lower than those obtained from 0.3% Mg-containing alloys. However, the 0.5% Mg alloy aged at 155 °C provides a maximum contribution to the strength of the base alloy containing 0.06% Mg which offered minimum contribution under the same aging condition (a base alloy in the as-cast condition was used as a reference alloy).
7. Quality index charts plotted for the three studied alloys revealed significantly different plots when the alloys were aged at 155 °C or 220 °C.
8. The fracture of fatigue-tested samples was found to be initiated by imperfections at the outer surfaces of the tested bars.

## Figures and Tables

**Table 1:** Chemistry of common secondary alloys (adapted after DYNCAST).

AA No.	Commercial Designation	Cu	Zn	Fe	Si	Mn	Mg	Cr	Ni	Ti
2081	108	3.5 4.5	1.0	0.9	2.5 3.50	0.50	0.10		0.35	0.25
296.1	B295.0/ B195	4.0 5.0	0.50	0.9	2.0 3.0	0.35	0.05		0.35	0.25
319.1	319	3.0 4.0	1.0	0.8	5.5 6.5	0.50	0.10		0.35	0.25
319.1 sr		3.0 4.0	1.0	0.9	5.5 6.5	0.50	0.25 0.40		0.35	0.25
355.1	355	1.0 1.5	0.35	0.50	4.5 5.5	0.50	0.45 0.6	0.25		0.25
356.1	356	0.25	0.35	0.50	6.5 7.5	0.35	0.25 0.40			0.25
A360.1	A360	0.6	0.40	1.0	9.0–10.0	0.35	0.45–0.6		0.50	
A380.1	A380	3.0–4.0	2.9	1.0	7.5–9.5	0.5	0.10		0.50	
B380.1		3.0–4.0	0.9	1.0	7.5–9.5	0.5	0.10		0.50	
383.1	383	2.0–3.0	2.9	1.0	9.5–11.5	0.5	0.10		0.30	
383–1		2.0–3.0	0.90	1.0	9.5–11.5	0.5	0.10		0.30	
384.1	384	3.0–4.5	2.9	1.0	10.5–12.0	0.5	0.10		0.50	
A384.1		3.0–4.5	0.9	1.0	10.5–12.0	0.5	0.10		0.50	
B390.1		4.0–5.0	1.4	1.0	16.0–18.0	0.5	0.50–0.65		0.10	0.20
A413.1	13	1.0	0.40	1.0	11.0–13.0	0.35	0.10		0.50	
C443.1	43	0.6	0.40	1.1	4.5 6.0	0.35	0.10		0.50	
706.1	603 Tern. 6	0.20	2.73 3.3	0.6	0.20	0.40 0.6	1.6 1.8	0.20 0.40		0.25
712.2	0712.0/	0.25	6.0 6.5	0.4	0.15	0.10	0.50 0.65	0.40 0.60		0.15 0.25
713.1	613	0.40 1.0	7.0 8.0	0.8	0.25	0.6	0.25 0.50	0.35	0.15	0.25

**Table 2:** Chemical composition of the as-used alloys (wt%).

Alloy	Si	Cu	Fe	Mg	Mn	Zn	Ti	Zn	Cr	Cr	Al
Experimental	9.5	3.5	-	-	-	-	-	--	--	--	Bal
A380.1	9.18	3.22	1.01	0.06	0.15	2.29	0.02	2.3	0.06	0.04	Bal

**Table 3:** Chemical composition of the as-cast industrial alloys.

Alloys *	Elements (wt%)					
	Cu	Fe	Mg	Mn	Zn	Ti
A	3.22	1.01	0.06	0.16	2.28	0.02
B	3.22	1.01	0.33	0.16	2.28	0.02
C	3.22	1.01	0.55	0.16	2.28	0.02

* Note: Alloys A, B, and C correspond to the same A380.1 alloy in Table 2, with different Mg levels.

**Table 4:** Reactions observed during the solidification of the present alloys (0.35 °C/s) using the thermal analysis technique.

Reaction No.	Temperature (◦C)				Reaction Details
	Experimental Alloy	Alloy A *	Alloy B	Alloy C	
1	580	572.7	572.9	570.4	Formation of α-Al network
2		567.7	567.7	564.6	Precipitation of β-Fe phase
3	563	563.8	561.9	560.6	Formation of (Al+Si) eutectic
4		536	539.5	537.6	Partial transformation of β-Fe → Al_8_Mg_3_FeSi_6_ phase
5		513.8	508.8	508.3	Precipitation of Mg_2_Si phase
6		475.5	509.13/480	505.1	Formation of Q-Al_5_Mg_8_Cu_2_Si_6_ phase
7	500	485	490	488	Formation of (Al + Al_2_Cu) eutectic
8	475	460.1	470.0	470.2	End of solidification

* Base alloy.

**Table 5:** Tensile properties of alloy A in the as-cast condition.

UTS (MPa)	YS (MPa)	%El
605	210	1.73

## Data Availability

Data will be made available upon request.
